# Genome-Wide Analyses and Functional Classification of Proline Repeat-Rich Proteins: Potential Role of eIF5A in Eukaryotic Evolution

**DOI:** 10.1371/journal.pone.0111800

**Published:** 2014-11-03

**Authors:** Ajeet Mandal, Swati Mandal, Myung Hee Park

**Affiliations:** Oral and Pharyngeal Cancer Branch, NIDCR, National Institutes of Health, Bethesda, Maryland, United States of America; University of British Columbia, Canada

## Abstract

The eukaryotic translation factor, eIF5A has been recently reported as a sequence-specific elongation factor that facilitates peptide bond formation at consecutive prolines in *Saccharomyces cerevisiae*, as its ortholog elongation factor P (EF-P) does in bacteria. We have searched the genome databases of 35 representative organisms from six kingdoms of life for PPP (Pro-Pro-Pro) and/or PPG (Pro-Pro-Gly)-encoding genes whose expression is expected to depend on eIF5A. We have made detailed analyses of proteome data of 5 selected species, *Escherichia coli*, *Saccharomyces cerevisiae*, *Drosophila melanogaster*, *Mus musculus* and *Homo sapiens*. The PPP and PPG motifs are low in the prokaryotic proteomes. However, their frequencies markedly increase with the biological complexity of eukaryotic organisms, and are higher in newly derived proteins than in those orthologous proteins commonly shared in all species. Ontology classifications of *S. cerevisiae* and human genes encoding the highest level of polyprolines reveal their strong association with several specific biological processes, including actin/cytoskeletal associated functions, RNA splicing/turnover, DNA binding/transcription and cell signaling. Previously reported phenotypic defects in actin polarity and mRNA decay of eIF5A mutant strains are consistent with the proposed role for eIF5A in the translation of the polyproline-containing proteins. Of all the amino acid tandem repeats (≥3 amino acids), only the proline repeat frequency correlates with functional complexity of the five organisms examined. Taken together, these findings suggest the importance of proline repeat-rich proteins and a potential role for eIF5A and its hypusine modification pathway in the course of eukaryotic evolution.

## Introduction

The eukaryotic translation factor, eIF5A, is a highly conserved and essential protein, but its mechanism in translation has remained enigmatic for decades (see recent review) [1] since its initial isolation from rabbit reticulocyte lysate [2]. eIF5A stimulates synthesis of methionyl-puromycin [2]–[4], a model assay for the first peptide bond formation. Yet, its role in translation initiation was questioned, as eIF5A could stimulate methionyl-puromycin synthesis with a preformed 80S initiation complex [4]. Increased polysomes in *S. cerevisiae* eIF5A mutants in the absence of functional eIF5A [5], [6], provided evidence that eIF5A is, in fact, involved in translation elongation rather than initiation. Furthermore, the fact that a rapid depletion of eIF5A in a yeast mutant strain caused a relatively modest inhibition in overall protein synthesis, led to a hypothesis that eIF5A is not involved in global translation, but stimulates translation of a subset of mRNAs [7].

eIF5A undergoes a novel posttranslational modification in which one specific lysine is converted to an unusual amino acid, hypusine, [N^ε^-(4-amino-2-hydroxybutyl) lysine] (see a review) [8]. In the first step, deoxyhypusine synthase (DHS) transfers the 4-aminobutyl moiety of the polyamine spermidine to an ε-amino group of a specific lysine (Lys 50 in human eIF5A) of the eIF5A precursor to form deoxyhypusine. The amino butyl side chain of this intermediate is subsequently hydroxylated by deoxyhypusine hydroxylase (DOHH). Hypusine synthesis occurs exclusively in this cellular protein [9] and is essential for eIF5A activity and cell proliferation [10]. Like eIF5A, DHS and DOHH are highly conserved in eukaryotes, suggesting their fundamental importance throughout eukaryotic evolution [10].

Elongation factor P (EF-P) is a bacterial ortholog that exhibits structural and functional analogy to eIF5A [11]–[14]. It is found in all eubacteria. EF-P was reported to stimulate synthesis of *N*-formylmethionyl-initiated peptides or *N*-formylmethionyl-puromycin. The crystal structures of EF-P domains I and II are superimposable on those of the archaeal initiation factor aIF5A [15] and are also similar to those of eIF5A. EF-P does not undergo hypusine modification as there is no homologous gene for DHS or DOHH in eubacteria [10]. Instead, the conserved lysine (Lys 34 in *E. coli* EF-P) that corresponds to the Lys modified to hypusine in eIF5A, is converted to beta-lysyl-hydroxy-lysine by a distinct posttranslational modification reaction, involving three enzymes, YjeK, YjeA and YfcM [16]–[19]. beta-lysylation of EF-P enhanced the activity of EF-P *in vitro* [17], [20].

During translation elongation, the ribosome catalyzes the synthesis of the peptide bond between the donor peptidyl-tRNA and the acceptor aminoacyl tRNA. However, not all peptide bonds are formed with equal efficiency, as certain amino acids are poor donors or acceptors. In particular, proline is ineffective as an acceptor as well as a donor and glycine is a poor acceptor in the peptidyl transferase reaction, causing the ribosome to stall. Recently, an important breakthrough was made by Ude et al. [21] and Doerfel et al. [22] on the role of EF-P in alleviating ribosome stalling. They independently demonstrated that EF-P could promote peptide bond formation at consecutive proline residues, such as PPP (Pro-Pro-Pro) or PPG (Pro-Pro-Gly), in *E. coli* or in a reconstituted *in vitro* translation system. Furthermore, Gutierrez et al. [23] reported similar activity of eIF5A in *S. cerevisiae*, including a model of eIF5A bound to 80S ribosome with its hypusine residue pointing to the peptidyl transferase center, supporting the notion that eIF5A has a critical role in the translation elongation of polyproline motifs. All living organisms contain either EF-P, or aIF5A or eIF5A and this factor(s) is one of the few universally conserved translation factors [24]. Furthermore, EF-P and aIF5A/eIF5A have analogous modifications. However, eIF5A and its modifying enzymes DHS and/or DOHH have become essential in eukaryotes, whereas EF-P and its modifying enzymes are not essential in bacteria.

The important, and unique, functional and structural roles of proline-rich motifs have been recognized in various cellular processes. Many different proline-rich regions occur widely in eukaryotic proteins (see reviews), [25]–[27]. Proline is an imino acid with its side-chain cyclized onto the backbone nitrogen. Three or longer consecutive prolines, or proline-rich regions containing proline as every third amino acid, can form a polyproline type II (PPII) helix, a unique left-handed helical structure with three residues per turn. Although all 20 amino acid residues occur in the PPII structure, proline has the strongest PPII propensity [28]. PPII often occurs at the edges of α-helices, in the domain linker regions, protein terminal regions and interaction interfaces. As the conformation of the PPII helix is restricted and is often exposed, the energy required for binding this helix is low. Highly specific, yet low affinity interactions involving the PPII helix occur in cellular processes requiring a transient and reversible recruitment or interchange of multiple proteins, such as initiation of transcription, RNA splicing, signaling cascades, and cytoskeletal rearrangements. Indeed, short linear proline-containing motifs have been recognized as targets of SH3, WW, EVH1 and GYF domains [26], [29] and proposed to play a crucial role in the assembly and regulation of intracellular signaling complexes.

The intriguing possibility of a connection between eIF5A and proline-rich proteins and their functional significance prompted us to investigate the occurrence of proline repeat motifs in the proteomes of 35 organisms representing six kingdoms of life, archaebacteria, eubacteria, protista, fungi, plantae and animalia. We made further detailed analyses of the complete proteomes of five representative species, *E. coli*, *S. cerevisiae*, and three metazoan species, *D. melanogaster*, *M. musculus* and *H. sapiens*. As PPP, and/or PPG, was identified as a ribosome stalling unit that is alleviated by EF-P [22] and eIF5A [1], [23], we have used these two motifs to probe the proteome databases. Our data clearly illustrate that the polyproline and PPG motifs have dramatically increased in number and frequency with biological complexity of organisms. Functional classification reveals enrichment of polyproline motifs in special ontology groups, including actin cytoskeletal function, RNA splicing/turnover, DNA binding and transcription and signal transduction pathways. Thus eIF5A and its hypusine modification pathway may have evolved along with certain eukaryote-specific, developmental functions dependent on proline repeat motifs.

## Materials and Methods

### Retrieval of proteome data

The data for protein sequences encoded by protein coding genes were downloaded from various genome databases through the websites or FTP (file transfer protocol) servers. We used the EcoGene 3.0 (http://bmb.med.miami.edu/ecodownload/dbtable) [30] and *Saccharomyces* Genome Database (SGD, http://www.yeastgenome.org/download-data/sequence) [31] for the retrieval of verified ORFs encoded protein sequence for *E. coli* and *S. cerevisiae*, respectively. The TrichDB (http://trichdb.org/common/downloads/Current_Release/) [32], PomBase (ftp://ftp.ensemblgenomes.org/pub/fungi/current/fasta/schizosaccharomyces_pombe/pep/) [33] and TAIR (ftp://ftp.arabidopsis.org/home/tair/User_Requests/) [34] were used for the complete proteins sequence data of *Trichomonas vaginalis*, *Schizosaccharomyces pombe* and *Arabidopsis thaliana* respectively.

The sequence datasets for proteins encoded by verified protein coding ORFs of *Ciona intestinalis* (Seasquirt), *Amphimedon queenslandica*, *Nasonia vitripennis*, *Drosophila melanogaster* (Fruitfly), *Anopheles gombiae*, *Caenorhabditis elegans*, *Xenopus laevis* (Clawed frog), *Danio rerio* (zebrafish), *Anolis carolinensis* (Anole lizard), *Gallus gallus* (Chicken), *Mus musculus* (Mouse), *Bos taurus* (Cow), *Homo sapiens* (Human) were fetched from Ensembl (release 75) BioMarts (http://www.ensembl.org/biomart/martview/) [35]. The complete proteome data of *Methanocaldococcus infernus*, *Pyrococcus abyssi*, *Thermococcus barophilus*, *Metalloshaera cuprina*, *Halophilic archaeon*, *Sulfolobus islandicus*, *Streptococcus pneumonia*, *Fusobacterium necrophorum*, *Yersinia pestis*, *Salmonella enterica*, *Giardia lamblia*, *Dictyostelium discoideum*, *Schizosaccharomyces cryophilus*, *Aspergillus flavus*, *Populus trichocarpa* (Cottonwood) and *Oryza sativa* (Rice) were downloaded from Ensembl website (http://useast.ensembl.org/index.html) [36]. In order to keep our analyses uniform we considered only the set of verified ORFs in each species that encode proteins including all the putative and uncharacterized proteins. After filtering out the proteins encoded by ORFs from pseudogenes and transposons, the data sets were sorted by selecting unique transcripts for each associated gene. The proteome was finalized by choosing and compiling the longest peptide sequence corresponding to each gene in an organism. UniProt [37] was used for the mapping of an entry to specific database identifier wherever required.

### Phylogenetic analysis of EF-P/aIF5A/eIF5A

The homologs of aIF5a, EF-P and eIF5A were identified by basic local alignment search tool (BLAST) by selecting the sequence with highest identity. The NCBI Reference Sequence identifiers (RefSeq IDs) are YP_003617054.1 (*M. infernus*), NP_126365.1 (*P. abyssi*), YP_004070806.1 (*T. barophilus*), YP_004410560.1 (*M. cuprina*), WP_014050471.1 (*H. archaeon*), YP_002839880.1 (*S. islandicus*), WP_000568638.1 (*S. pneumoniae*), WP_005957090.1 (*F. necrophorum*), WP_000257280.1 (*E. coli*), AAS60780.1 (*Y. pestis*), WP_023785440.1 (*S. enterica*), ADE62353.1 (*T. vaginalis*), XP_639978.1 (*D. discoideum*), EFO63693.1 (*G. lamblia*), NP_010880.3 (*S. cerevisiae*), EPY53783.1 (*S. cryophilus*), NP_594457.1 (*S. pombe*), XP_002374390.1 (*A. flavus*), XP_006455784.1 (*A. bisporus*), XP_002325136.1 (*P. trichocarpa*), NP_172848.1 (*A. thaliana*), CAB96075.1 (*O. sativa*), XP_002127566.1 (*C. intestinalis*), XP_003384319.1 (*A. queenslandica*), XP_001607976.1 (*N. vitripennis*), AF187730_1 (*D. melanogaster*), XP_564212.2 (*A. gambiae*), NP_495807.1 (*C. elegans*), NP_001080536.1 (*X. laevis*), NP_998350.1 (*D. rerio*), XP_003218182.2 (*A. carolinensis*), NP_990863.1 (*G. gallus*), (*M. musculus*), XP_005201728.1 (*B. taurus*), NP_001961.1 (*H. sapiens*). In case of eukaryotes, one of the isomers of eIF5A was selected with highest identity score with human *EIF5A* gene. All the sequences were aligned by ClustalW [38] using default parameters and the evolutionary relationship was inferred using the Neighbor-Joining method [39]. Evolutionary analyses and the construction of phylogenetic tree were conducted in MEGA6 [40].

### Identification of proteins containing PPP or PPG motifs and their quantitation

**Figure 1:**
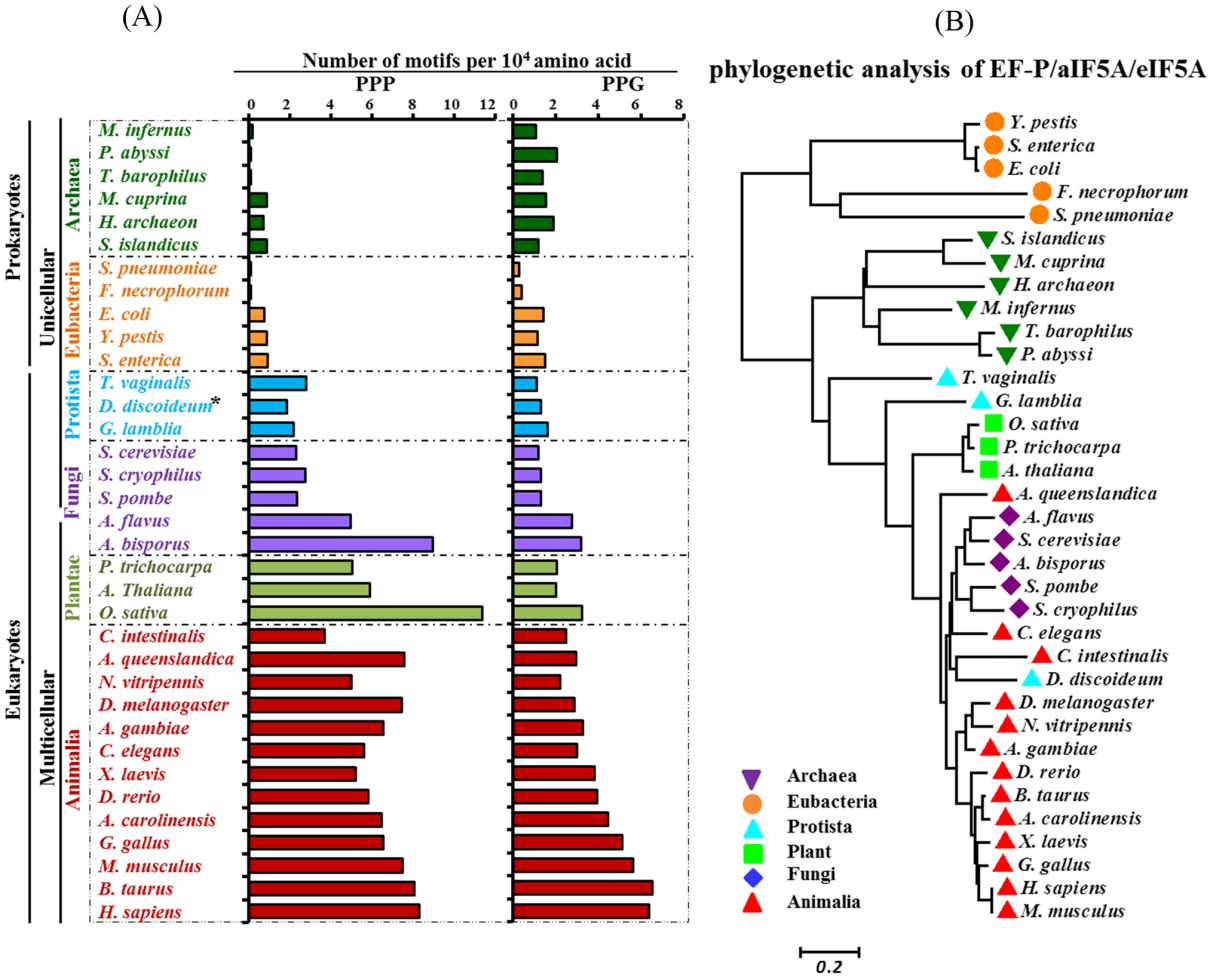
The frequencies of PPP and PPG motifs in the complete proteomes of 35 organisms from six kingdoms of life and a phylogenetic analysis of EF-P/aIF5A/eIF5A. (A) The complete proteomes of 35 species were retrieved from multiple databases as described under Materials and Methods. The PPP and PPG frequencies are indicated as numbers per 10,000 amino acids. The total numbers of PPP motif, PPG motif, ORFs and amino acids in the whole proteome of each organism as well as the frequencies of PPP and PPG motifs (calculated by the division of total PPP or PPG motifs by total amino acids) are listed in Table S1. An asterisk (*) indicates a unicellular eukaryote that can develop into a multicellular organism. (B) An evolutionary relationship of EF-P/aIF5A/eIF5A homologs among 35 different organisms is represented by a phylogenetic tree.

The proteins containing proline triplets or PPG motifs were identified by regular expression in Perl (practical extraction and report language). All the complete proteome datasets were examined strictly for the presence and the total counts of three consecutive prolines (PPP) or PPG without overlaps in each protein. The number of PPP units in each protein was determined as the whole number after division of the consecutive proline length by 3. For example, 3, 4 and 5 consecutive prolines were all counted as 1 PPP unit and 6, 7, and 8 consecutive prolines as 2 PPP units. To determine the number of genes encoding ≥1, ≥2, and ≥3 PPP or PPG, the number of PPP or PPG units was calculated for each gene. Groups of genes encoding one or more (≥1), two or more (≥2), three or more (≥3) PPP units or PPG motifs were compiled for each species. The data for PPP and PPG frequency in the total proteome of the 35 organisms are shown in Fig. 1A and Table S1. The genes encoding ≥1 PPP or PPG from each selected species are listed in Table S2–Table S17.

### Identification of orthologous genes and generation of ortholog pools

We used InParanoid8 [41] for the identification of orthologous genes from five species, *E. coli*, *S. cerevisiae and D. melanogaster*, *M. musculus* and *H. sapiens*. Inparanoid8 is mainly an eukaryotic ortholog database; however, it includes *E. coli* as the only representative from prokaryotes. We chose this database over other tree-based methods for the current study because it is a BLAST-based approach and uses the pairwise similarity scores to achieve the maximum sensitivity in obtaining an orthologous cluster. In InParanoid8, the genome datasets are precomputed for the ortholog cluster assignment and the data source are available. To obtain the best matching ortholog pairs, we only selected the orthologs with Inparalog score equal to 1.0 in each cluster and filtered out all others. In view of above parameters, depending on the commonly shared orthologous genes at different levels of evolutionary hierarchy, we have generated 4 different pools. To generate pool 1, the pairwise comparisons of *E. coli* orthologs with other four species *S. cerevisiae*, *D. melanogaster*, *M. musculus* and *H. sapiens* were retrieved from FTP server (http://inparanoid.sbc.su.se/download/8.0_current/Orthologs_other_formats/E.coli/) and the commonly shared *E. coli* orthologs in each comparison were listed as pool 1 for *E. coli*. In a similar manner the pool 1 for *S. cerevisiae*, *D. melanogaster*, *M. musculus* and *H. sapiens* were prepared. The *S. cerevisiae* orthologs compared to *D. melanogaster*, *M. musculus* and *H. sapiens* were obtained (http://inparanoid.sbc.su.se/download/8.0_current/Orthologs_other_formats/S.cerevisiae/) and the common genes were listed as pool 2 for *S. cerevisiae*. In the same way the pool 2 for *D. melanogaster*, *M. musculus* and *H. sapiens* were prepared. The *D. melanogaster* common orthologs in pairwise lists (http://inparanoid.sbc.su.se/download/8.0_current/Orthologs_other_formats/D.melanogaster/) compared to *M. musculus* and *H. sapiens* were listed as pool 3 for *D. melanogaster*. Similarly, pool 3 for *M. musculus* and *H. sapiens* were generated. Pool 4 for mouse and human consists of the orthologs (http://inparanoid.sbc.su.se/download/8.0_current/Orthologs_other_formats/H.sapiens/) shared among these species. The frequencies of polyproline and PPG motifs were computed using Perl.

### Computation of protein lengths and amino acid occurrence

The exact number of amino acids in each protein from each of the complete proteomes of five different species was computed by Perl. The total number of each twenty different amino acids and the occurrences of triplets of each amino acid in different species were calculated using regular expression in Perl. The frequencies of each amino acid and their triplets were then compared across all five species.

### Gene ontology (GO) classification

The functional categories for the selected *S. cerevisiae* genes were determined by combining the GO annotation data available on SGD [31] based on specific biological process and molecular function of a gene. The 76 genes encoding ≥2 PPP units are listed in descending order with the gene encoding the highest polyproline as the first (Table S18); the gene name, description and actual number of proline repeats (not PPP units) are given. The ortholog genes (257 *S. cerevisiae* genes) are listed in Table S19. Human genes encoding PPP or PPG motifs at high frequency (>0.01, >10 fold over that of the total proteome) (Table S20 and Table S21) were assigned to 7 major functional categories using PANTHER (http://pantherdb.org/) [42].

## Results and Discussion

### Occurrence of PPP and PPG motifs in various organisms and phylogenetic analysis of EF-P/aIF5A/eIF5A

We first searched the genome databases of 35 organisms representing the six kingdoms of life, including archaea, eubacteria, protista, fungi, plantae and animalia, for the occurrence of PPP and PPG motifs (Fig. 1A, Table S1). We assigned PPP unit numbers for polyproline stretches longer than 3 prolines (as a whole number after division of the consecutive proline number by 3) and determined the total number of PPP and PPG units in each proteome. We then calculated the frequency of their occurrence by dividing the total number of the PPP or PPG units with the total number of amino acids in each proteome. It is apparent that there is a general increase in the frequencies of PPP and PPG motifs in higher organisms (Fig. 1A). Although a certain degree of variation is seen in different organisms of the same kingdom, the PPP frequencies are generally quite low (0.1–1.0 per 10,000 amino acids) in the proteomes of archaea and eubacteria, but are increased in higher organisms, *i.e.* fungi, plant and animals (2.3–11 per 10,000 amino acids). The PPG frequencies tend to be higher in those of multicellular fungi, plants and animals (2.0–6.5 per 10,000 amino acids) than in archaea, eubacteria, protista and unicellular fungi (0.25–2 per 10,000 amino acids). The frequencies of PPP and PPG motifs are, in general, higher in the multicellular eukaryotes of fungi, plants and animals, than in unicellular organisms of fungi, protista, eubacteria and archaea. Among the vertebrates, PPP and PPG frequencies appear to increase with the functional complexity of the organisms. However, these frequencies did not correlate with the ORF numbers of the organisms (Table S1).

The phylogenetic tree based on homologs of EF-P/aIF5A/eIF5A suggests an evolutionary relationship between different taxa (Fig. 1B). Eukaryotes forms a separate lineage from those of archaea and eubacteria and its members share a considerable degree of homology, which further extends to even higher degree among animalia. This finding goes along with the increasing frequencies of PPP and PPG motifs with the complexity of eukaryotes and shows some degree of correlation between the phylogeny of this factor and the PPP/PPG frequencies in various organisms.

### Increase in the occurrence of PPP and PPG motifs in proteins during eukaryotic evolution

**Figure 2:**
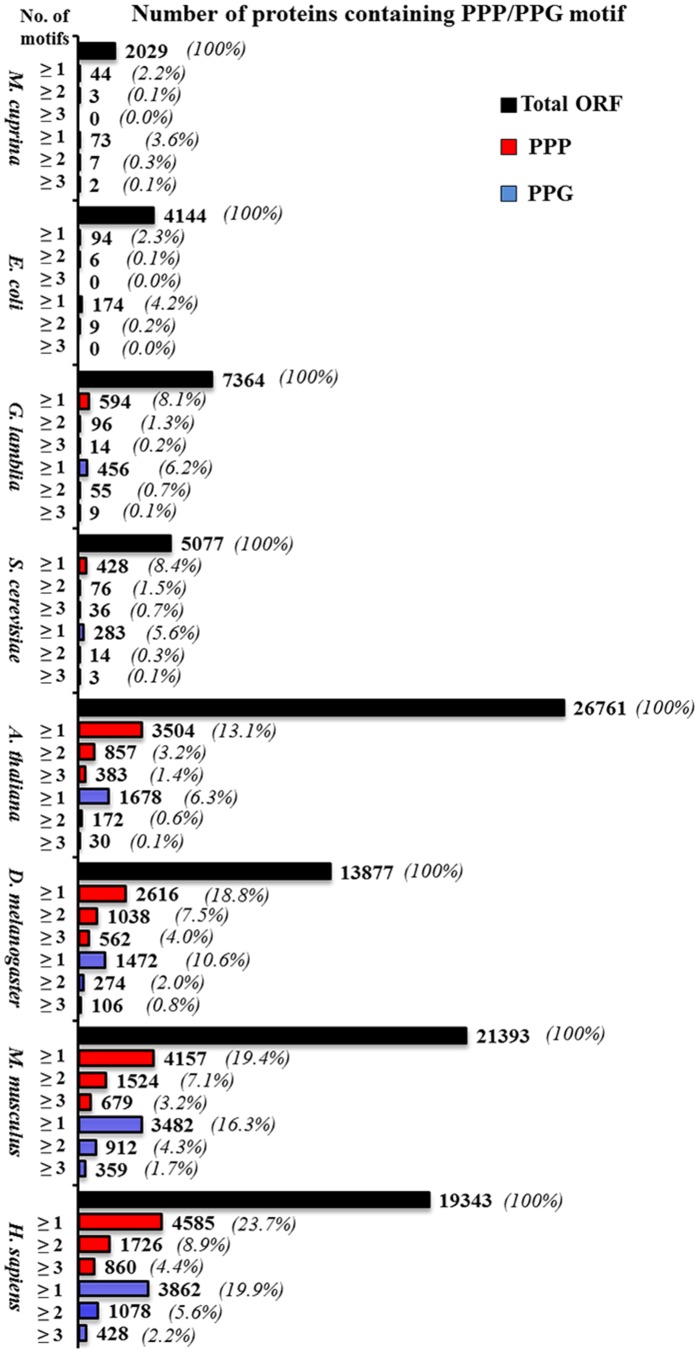
Comparison of the number and percentage of proteins containing PPP or PPG motifs in *M. cuprina*, *E. coli*, *G. lamblia*, *S. cerevisiae*, *A. thaliana*, *D. melanogaster*, *M. musculus*, and *H. sapiens*. Proteins containing ≥1, ≥2 and ≥3 PPP or PPG units were compiled as described under Materials and Methods and these groups are indicated by unit numbers, ≥1, ≥2 and ≥3 on the Y-axis along with the organism name. The number of proteins in these groups, or in the total pool, is indicated as bars with a value on the right side of each bar and the percent in brackets. The complete lists of genes (name, description, amino acid length and number of PPP or PPG units) encoding proteins with ≥1 PPP or PPG units for each species are provided as Supporting Information (Table S2–Table S17).

Of the 35 organisms displayed in Fig. 1, we first selected 8 organisms, at least one from each kingdom, *M cuprina*, *E. coli*, *G lamblia*, *S. cerevisiae*, *A thaliana*, *D. melanogaster*, *M.musculus and H. sapiens* for detailed analyses. Proline repeats occur in various lengths in different proteomes, but triple proline (3P) alone comprises the majority of polyprolines in all the proteomes (Table 1). Whereas triple proline is by far the major polyproline (91%) in *E. coli*, longer proline stretches (4 P to >10 P) increase in eukaryotes. The number of polyproline motifs (3P to 8P), as well as the total number, showed a consistent increase in more complex organisms (lowest in bacteria and highest in human). We then compared both the number and the percent of proteins containing PPP or PPG motifs (≥1, ≥2, and ≥3 units) in the eight organisms. As shown in Fig. 2, both the number and the percent of proteins containing PPP or PPG motifs have increased remarkably from prokaryotes to high eukaryotes. For instance, the percentage of proteins with ≥1 PPP unit is 2.2, 2.3, 8.1, 8.4, 13.1, 18.8, 19.4 and 23.7%, and that for those with ≥1 PPG is 3.6, 4.2, 6.2, 5.6, 6.3, 10.6, 16.3 and 19.9%, respectively, for *M. cuprina*, *E. coli*, *G. lamblia*, *S. cerevisiae*, *A. thaliana*, *D. melanogaster*, *M. musculus* and *H. sapiens*. The percent increased by approximately 10 and 5 fold, respectively, for PPP- and PPG-containing proteins in human over the bacteria. Moreover, there are no proteins containing proline repeats corresponding to ≥3 PPP or ≥3 PPG units in *E. coli*, whereas in mammals, hundreds of proteins contain ≥3 PPP or ≥3 PPG units (as many as 39 PPP or 68 PPG units per protein in human).

**Table 1:** Number of consecutive proline repeats in the eight proteomes.

Species	3P	4P	5P	6P	7P	8P	9P	10P	>10 P	Total number	3P as % of total
*M. cuprina*	43	3	1	0	0	0	0	0	0	47	91.5
*E. coli*	90	6	2	0	0	1	0	0	0	99	90.9
*G. lambia*	603	77	16	3	3	1	0	0	0	703	85.8
*S. cerevisiae*	442	60	36	12	3	3	4	1	2	563	78.5
*A. thaliana*	3751	1481	457	161	78	53	24	23	20	6048	62.0
*D. melanogaster*	3605	834	328	159	91	39	19	5	15	5095	70.8
*M. musculus*	5495	1189	386	188	95	57	47	31	58	7546	72.8
*H. sapiens*	6027	1330	487	218	127	66	42	27	41	8365	72.1

Total proteome of each species was searched with XPnX (n = 3 to >10) where X is other than proline.

### Average protein length and frequency of the PPP or PPG motif increase in higher organisms

**Figure 3:**
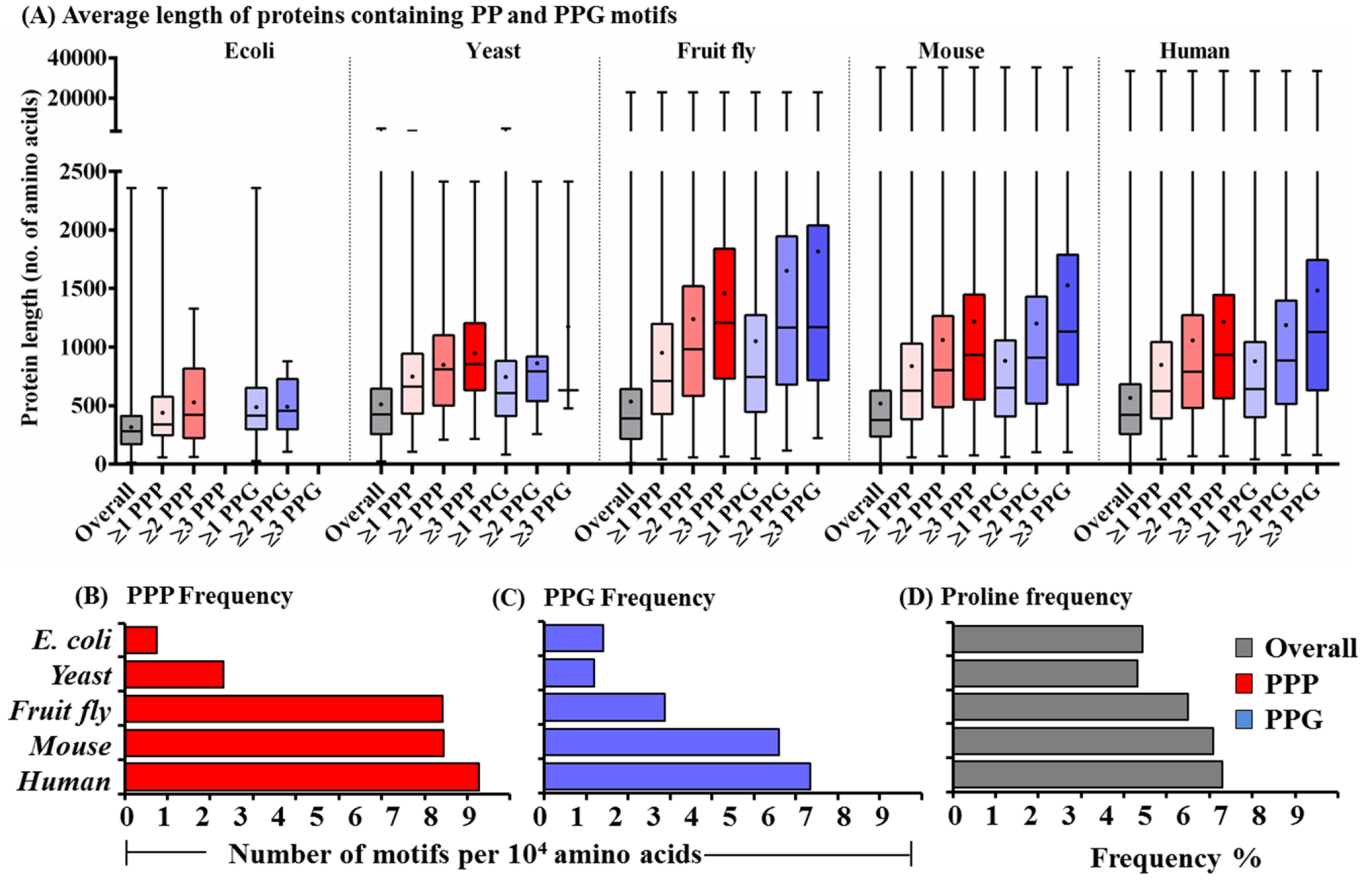
The distribution of protein lengths and the frequencies of PPP, PPG and proline in proteomes of the five species. (A) Protein length is expressed as the number of amino acid residues per protein. The distribution of protein lengths of total proteins and of those containing ≥1, ≥2, and ≥3 PPP or PPG units are represented by box and whisker plot. The boxes indicate the 25^th^ and 75^th^ percentile; horizontal line in the box is plotted at the median and the whiskers ranges from minimum length to maximum. The mean is shown by a black dot inside the boxes. Frequencies of (B) PPP, (C) PPG and (D) proline were calculated by division of total number of PPP or PPG units and of proline with total amino acid numbers in each proteome and the actual values are listed in Table 2.

We then analyzed the distribution of the length of proteins (the number of amino acid residues) of five most commonly studied organisms, *E. coli*, *S. cerevisiae*, *D. melanogaster*, *M. musculus* and *H. sapiens*, in a total proteome or in groups containing ≥1, ≥2, or ≥3 PPP or PPG units. The data in Fig. 3A shows protein size ranges (whiskers) and distribution of the majority of proteins (the boxes indicate those within 25^th^ and 75^th^ percentiles). The largest protein lengths (indicated by top end of whiskers) are 2358, 4910, 22949, 35213, and 33420 amino acids, and the average length of total proteins in each proteome, 317, 510, 535, 519 and 566 amino acid residues per protein (dots in the colored boxes) and the median values, 282, 426, 392, 376 and 422 amino acids (horizontal lines in the boxes), respectively, for *E. coli*, *S. cerevisiae*, *D. melanogaster*, *M. musculus* and *H. sapiens*. The increases in protein sizes may be due to the fusion of single-functional or single-domain proteins into multi-functional and multi-domain proteins during eukaryotic evolution [43]. In the *E. coli* proteome, there are only small differences in the average protein length in groups containing ≥1 or ≥2 PPP or PPG. However, in the eukaryotes the proteins (on average) are consistently longer in the groups with higher PPP or PPG content (Fig. 3A). For instance, in the groups containing high proline repeats (≥3 PPP or ≥3 PPG), the average protein lengths are over twice those of the total proteins in the fly, mouse and human proteomes. The data imply that, in general, those proteins containing high proline repeats are longer and newly evolved.

As there are proteins containing multiple PPP or PPG units, and as proteins tend to be longer in higher organisms, the percentage of genes in Fig. 2, is not an accurate representation of the abundance of these motifs. Therefore, we determined the frequency of their occurrence (Table 2). The frequency of PPP (Fig. 3B) was estimated to be 0.76×10^−4^, 2.31×10^−4^, 7.43×10^−4^, 7.45.31×10^−4^, and 8.28×10^−4^, and that of PPG (Fig. 3C) was 1.39×10^−4^, 1.17×10^−4^, 2.87×10^−4^, 5.60×10^−4^, and 6.35×10^−4^, respectively, for the total proteome of *E. coli*, *S. cerevisiae*, *D. Melanogaster*, *M. musculus*, and *H. sapiens*. The polyproline frequency increased ≥3 fold in the yeast over the bacteria, and 3.2 fold in the fly over the yeast, implying the emergence of new biological functions associated with polyproline motifs in the yeast and multicellular eukaryotes. On the other hand, the PPG frequency did not increase in the yeast over that of the bacteria, but increased 2.5 fold in the fly over the yeast and 2.0 fold in the mouse over the fly suggesting their importance in development of multicellular eukaryotes and mammals. We also determined the proline usage frequency in the different proteomes. The difference in the frequency of proline usage (1.4 fold in human over that in bacteria) (Fig. 3D) is relatively small compared to that of PPP (11 fold, human to *E. coli*) or PPG (4.6 fold, human to *E. coli*). Thus, the increased PPP or PPG frequency is not attributable to a general increase in proline usage in the eukaryotic proteome.

**Table 2:** Number and frequencies of PPP, PPG or proline in the five proteomes.

Species	Total number of PPP units	Total number of PPG units	Total number of Prolines	Total number of amino acids	PPP frequency/10^4^ amino acids	PPG frequency/10^4^ amino acids	Proline frequency %
*E. coli*	100	183	58315	1312586	0.76	1.39	4.44
*S. cerevisiae*	597	304	111620	2587553	2.31	1.17	4.31
*D. melanogaster*	5512	2127	408685	7421447	7.43	2.87	5.51
*M. musculus*	8282	6218	676678	11111878	7.45	5.60	6.09
*H. sapiens*	9097	6978	693470	10993264	8.28	6.35	6.3

Total number of PPP or PPG units in each proteome was calculated as described in Materials and Methods.

### Increases in average length and the frequency of PPP and PPG in orthologous proteins of higher organisms

**Figure 4:**
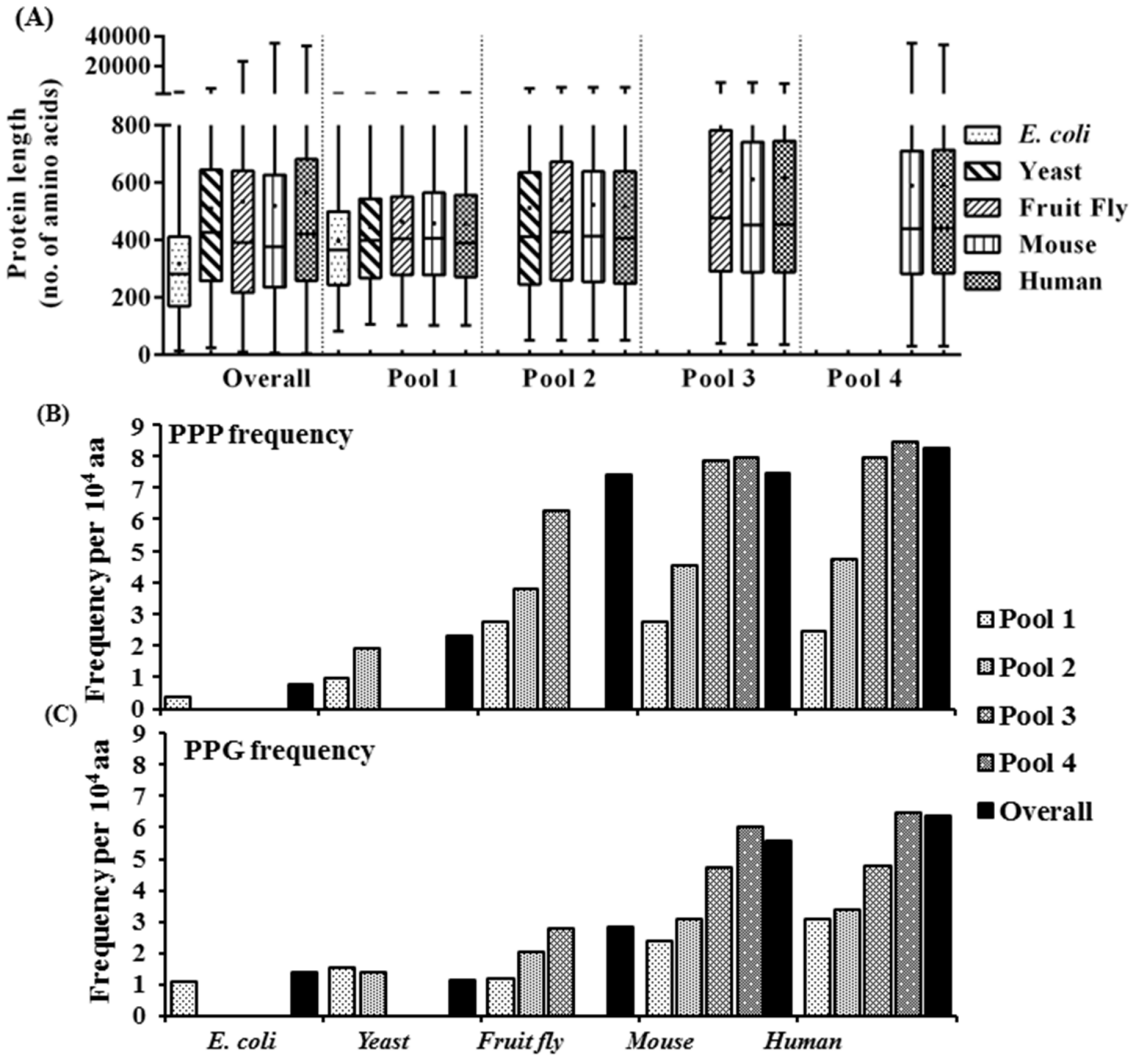
Comparison of (A) average protein length, the frequency of (B) PPP and of (C) PPG in the total proteome and in orthologous protein pools. The orthologous protein pools were generated from each species: pool 1, those shared by all five species; pool 2, those shared by four eukaryotes; pool 3, those shared by the fly, mouse and human; pool 4, those shared by mouse and human and the values of ortholog pools were compared with those of the total proteins pool in each species. For each species, pool numbers are indicated, and T denotes total pool. The distribution of protein lengths is shown by box and whisker plots and the frequencies of PPP and PPG were determined as in Fig. 3.

We examined average protein length and frequency of the proline repeat motifs in orthologous proteins in four different pools: pool 1 from all five species, pool 2 from four eukaryotes, pool 3 from fly, mouse and human and the pool 4 from mouse and human (Fig. 4). Of the four pools, the orthologs in pool 1, commonly shared in the five species, represent the oldest proteins, and those in pool 4, of mouse and human proteins, predominantly the newest proteins. Compared to the overall pool, the average protein lengths in the same ortholog pools are quite similar among different species (Fig. 4A). For example, the average protein length for pool 1, 2, 3 and 4 ranges between 398–463, 511–540, 613–642 and 591–594 amino acids, respectively, whereas that in the overall pool ranges between 317–566 amino acids. For the same species, the average length in the newer ortholog pool was higher than that in the older pools. Furthermore, the maximum lengths of proteins increased dramatically in the newer pools. These findings suggest an inverse relationship between protein age and length and are in line with the data of Wang et al. [44] that showed conservation of the length of the orthologous proteins of five eukaryotes and the protein length increase in the newly derived proteins.

The PPP and PPG frequencies also showed a general increasing trend in higher organisms (Fig. 4B and Fig. 4C) in the orthologous pools as those of the total pools. However, the frequencies of both motifs increased less in the older protein pools 1 and 2 compared to those of the total pools, consistent with the notion that old proteins contain less PPP or PPG than newly evolved proteins. In the case of the pool 4, the PPP frequency was even higher than that in the total pool in mouse and human proteomes. In these two proteomes, there were large increases in the PPP frequencies in pool 2 over pool 1, and in pool 3 over pool 2, while the frequencies in pools 3 and 4 were quite similar (Fig. 4B). On the other hand, there is a jump in PPG frequency in pool 3 relative to pool 2 and also in pool 4 compared to pool 3 (Fig. 4C), signaling a role for the PPG motif in the development of multicellular eukaryotes and mammals.

### Comparison of the frequencies of proline triplets and other amino acid triple repeats

**Figure 5:**
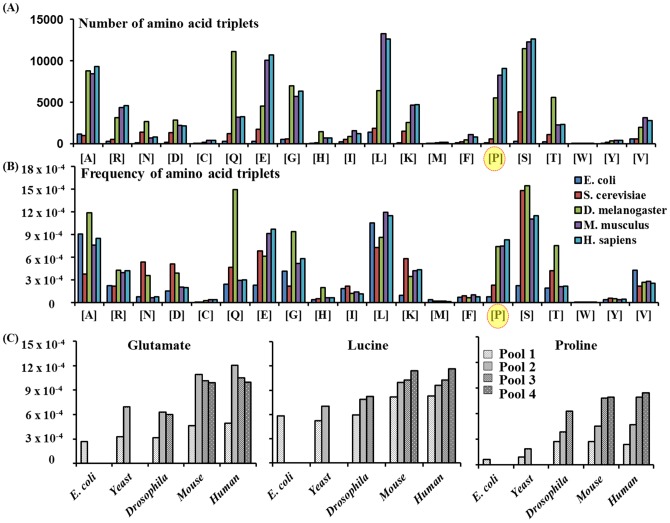
Number and frequency of tandem repeats of 20 individual amino acids in five proteomes and orthologous protein pools. (A) Amino acid repeats (≥3 amino acids) were estimated as triplet units. For repeats longer than 3 amino acids, the number of triplet units was assigned as the whole number from division of the consecutive amino acids with 3, as was done for PPP. (B) The frequency of each amino acid triplet unit was determined by division of the total number of triplet units with the total number of amino acids in each proteome listed in Table 2. (C) The frequencies of tandem repeats of Glu, Pro and Leu in four orthologous protein pools used in Fig. 4.

Tandem repeats of various amino acids occur widely in living organisms [45]. In order to determine whether the occurrence of the tandem repeats of amino acids other than proline have also increased during evolution, we searched the five proteomes for tandem repeats (≥3) of each amino acid (Fig. 5) using triplet as a unit in the same way as was applied to estimate polyproline units. Triple repeats of Ala, Gln, Glu, Gly, Leu, Pro and Ser are abundant in three metazoans whereas those of Tyr, Trp, Phe, Met and Cys are rare in all the proteomes. The total number of triplet units of Ala, Arg, Glu, Leu, Lys, Pro and Ser appeared to increase with evolution (Fig. 5A). However, for these and most other repeats, the frequencies (after calibration against each proteome size) varied randomly and inconsistently in different species (Fig. 5B). Only the Pro triplet frequency displayed consistent increases in higher organisms. We also examined the frequencies of all the other amino acid repeats in the four ortholog pools used in Fig. 4 (Fig. S1). Although frequencies of tandem repeats of Glu, Pro and Leu showed an increasing trend in more complex organisms, the frequency increases were much smaller (Leu repeat, <2 fold in human over *E. coli*) or inconsistent (Glu repeats), compared to those of Pro repeats (Fig. 5C). Similar increases in proline repeats have also been reported from other analyses of proteomes of eukaryotic species (human, fly, worm, yeast, weed) [46] or of orthologous proteins in rat, mouse and human [47]. Of all the amino acid repeats, the proline repeats exhibited the highest conservation in the three mammalian species examined [47] underscoring their biological relevance. The role of other amino acid repeats in evolution is not known. However, natural selection may have contributed to the accumulation of amino acid tandem repeats, as the conservation of coded amino acid repeats was much higher than that of non-coding repeats during vertebrate evolution [48]. Thus, it is likely that enrichment of polyproline motifs also occurred from natural selection in metazoans.

### Functional significance of the proline-rich motifs: Association of proline repeat-rich proteins with newly derived cellular mechanisms in eukaryotes

**Figure 6:**
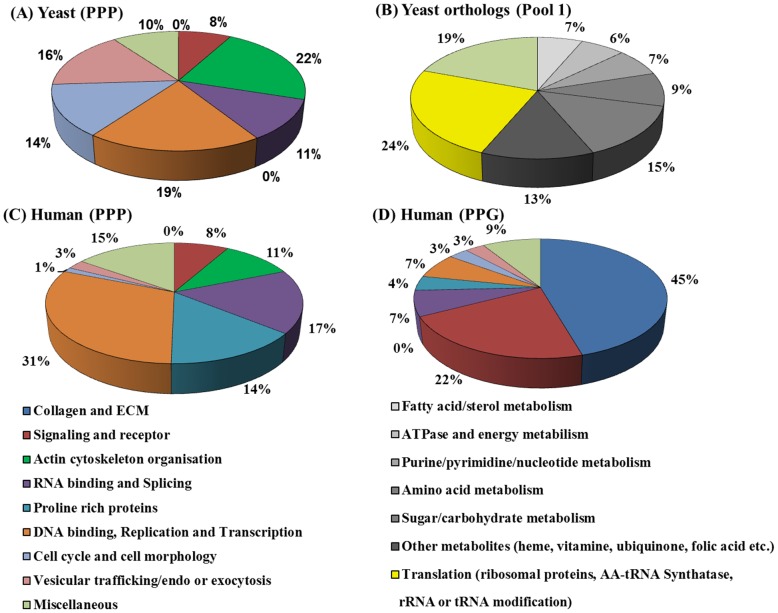
Functional ontology diagrams of *S. cerevisiae* genes encoding ≥2 PPP units, or yeast orthologous genes and of human genes encoding PPP or PPG motifs at highest frequency (>0.01). (**A**) 76 *S. cerevisiae* genes encoding highest level of polyproline (listed in Table S18) were classified based on the GO annotations available on SGD. (**B**) 257 *S. cerevisiae* orthologous genes (pool 1, genes common from *E. coli* to human, listed in Table S19) were classified as in (A). (C) 148 human genes encoding PPP at high frequency (>0.01) listed in Table S20 and (**D**) 79 human genes encoding PPG at the frequency (>0.01) listed in Table S21 were functionally classified.

We performed an ontology classification of the 76 *S. cerevisiae* genes encoding the highest proline repeat motifs (>2 PPP units) (Fig. 6A, Table S18) and compared with that of a pool of yeast orthologous genes commonly shared in the five species (Fig. 6B, Table S19). The PPP frequency of this group of genes is 0.0038, 16.6 fold higher than that of the total *S. cerevisiae* genes. These yeast genes encoding high polyproline repeats are mainly involved in six cellular processes specialized in eukaryotes: actin cytoskeleton-associated function (22%), DNA binding/replication/transcription factors (19%), RNA splicing/processing (11%), signal transduction/protein kinase (8%), vesicular trafficking/endocytosis/exocytosis (16%) and cell wall/cell cycle/morphology/budding (14%) (Fig. 6A). The importance of polyproline motifs in the actin cytoskeleton is also evident from the top five genes encoding the highest level of polyprolines, VRP1 (Proline-rich actin associated protein, 9P, 8P, 6P, 4×5P, 3×4P, 2×3P), LAS17 (Actin assembly factor, 6P, 8× 5P), AIM3 (Protein that inhibits barbed-end actin filament elongation, 5P, 2× 4P, 6× 3P), BNI1 (formin, 12P, 10P, 5P) and BNR1 (formin, nucleates the formation of linear actin filaments, 11P, 8P, 2×3P). No *E. coli* ortholog is found for the 76 polyproline-rich yeast proteins, suggesting that all of them originated in eukaryotes. It is indeed quite remarkable that the proteins most enriched in polyproline motifs segregate into several major functional groups that distinguish eukaryotes from prokaryotes. In contrast, *S. cerevisiae* orthologs commonly shared from *E. coli* to human display a functional distribution quite different from that of the polyproline-rich proteins (Fig. 6B). The PPP frequency of *S. cerevisiae* pool 1orthologs (257 genes, Table S19), 0.0001 is much lower than that of the total yeast proteome (2.3 fold less than the total, and 38 fold less than the PPP-rich group) and represent the most basic cellular functions conserved from bacteria to human: 57% of the orthologs are annotated in various cellular metabolic pathways, including those of carbohydrate, amino acids, fatty acids, nucleotides, energy and various other components. 24% of them are involved in the translation machineries of cytoplasm and mitochondria, including ribosomal proteins, aminoacyl tRNA synthetases and enzymes modifying rRNA or tRNA. In fact, 38% of the orthologous proteins are localized in mitochondria, suggesting their bacterial origins.

Functional specificities of polyproline-rich proteins corroborate certain phenotypes of *S. cerevisiae* eIF5A mutant strains [49], [50]. Under restrictive conditions where mutant eIF5A is degraded or its activity impaired, various defects were observed in mRNA turnover, actin cytoskeleton, cell polarity, cell wall integrity, cell cycle and budding, but the precise role of eIF5A in these defects was not clear. Considering the strong association of polyproline-encoding genes with some of these functions, it is tempting to speculate that the mutant phenotypes are due to decreased translation of these polyproline-rich proteins in the eIF5A mutant strains. Defects in fertility and polarized cell growth due to deficiency of polyproline-rich formins in a yeast eIF5A mutant strain supports this notion [51]. An independent study is underway to identify, by a proteomics approach, cellular proteins whose levels are altered upon eIF5A deficiency.

The functional distribution of human proteins richest in polyproline motifs (148 genes in Table S20, frequency >0.01, >10 fold higher than average PPP frequency), (Fig. 6C) show a similar pattern to that of *S. cerevisiae*, in that a large percent of these proteins are involved in DNA binding and transcription (31%) actin cytoskeleton (11%), and RNA processing, splicing and metabolism (17%) and signaling/ligand/receptor (8%). In addition, approximately 14% of these proteins are proline-rich proteins, mostly those important in salivary function. Human proteins richest in the PPG motif (79 genes in Table S21, frequency >0.01, >15 fold higher than average PPG frequency) (Fig. 6D) consist of structural proteins (collagen and collagen-like, extracellular matrix proteins, 45%), and those involved in signaling/receptors (22%), RNA splicing/processing (7%) and DNA binding/transcription (7%). In the case of human proteins, differences are observed between the high PPP and high PPG groups. Actin/cytoskeleton-related functions and DNA binding/transcription are highly associated with polyproline motifs, whereas extracellular matrix structural proteins, signaling molecules and receptors are enriched in PPG motifs. Thus, both PPP- and PPG-rich proteins seem to serve important roles during metazoan development.

## Conclusions

The pronounced increases in the proline repeat motifs in higher organisms and predominant association of PPP- and PPG-rich proteins with several eukaryotic developmental processes strongly suggest the importance of polyproline motifs in eukaryotic evolution. Of all the amino acid repeats, consecutive proline repeats have unique structural features to form the poly-L-proline type II (PPII) helix. While PPII is a structural element of fibrillar proteins, *e.g.* collagen, more importantly, it holds functional significance in protein-protein and protein-nucleic acid interactions. The PPII helix is often located in intrinsically disordered regions and affords a highly specific, but low affinity binding, favorable in dynamic complexes involved in *e.g.* actin cytoskeleton regulation, RNA splicing, transcription and signal transduction. Although the PPII helix is not limited to proline-containing peptides, it is a dominant conformation in proline-rich regions in proteins. Evolutionary conservation of the PPII conformation, particularly surrounding intrinsically disordered phosphorylation sites, underscores their functional significance [52]. Many proline-rich regions of the PPII helix have been identified, including PXXP, PPxY, PxPPx, (XP)n, (XPY)n (where X or Y denotes any amino acid) in addition to polyproline stretches (Pn) and these motifs serve as specific ligands to various domains of the signaling molecules SH3, WW, EVH1, and to profilin. Consecutive polyproline repeats show the highest conservation among all amino acid repeats. Furthermore, the importance of proline repeats has been established experimentally in many cases by mutagenesis. Many proteins identified here using PPP or PPG as probes may contain PPII helices or other proline-rich regions and thereby may share similar functional classifications previously recognized for PPII motifs. In view of the functional role of PPII helices in protein-protein and protein-nucleic acid interactions critical in the development of multicellular organisms, it is not surprising that we also find a marked rise in the proline repeat motifs during metazoan evolution. It is also noteworthy that the abundance of polyproline recognition domains, such as SH3, WW, and EVH1, has increased drastically in human over yeast [29].

Furthermore, the remarkable increases in the frequencies of PPP and PPG motifs in higher organisms suggest a potential role for eIF5A in eukaryotic evolution, as eIF5A is known to promote peptide bond formation at proline repeat sequences. Although EFP also facilitates Pro-Pro peptide bond formation and is activated by a posttranslational modification, the two proteins differ in their essentiality. Unlike eIF5A, EFP is not an essential protein, nor are its modification enzymes, YjeA, YjeK, and YfcM. Mutants with deletion of *efp*, *yjek* or *yjea* are impaired in bacterial virulence and exhibit growth defects only under certain stress conditions [19]. The nonessential nature of this factor may reflect the fact that bacteria contain a small number of proteins containing polyprolyl motifs. In contrast to EFP, eIF5A and deoxyhypusine synthase are essential in eukaryotes. The requirement for deoxyhypusine hydroxylase may depend on the organism. A *S. cerevisiae* DOHH null strain is viable [53], suggesting that the deoxyhypusine-containing eIF5A intermediate can support growth in yeast. In contrast, this enzyme is required for development of multicellular eukaryotes, *e.g. C. elegans* [54], *D. melanogaster* [55] and mouse [56]. Considering the stringency in the requirement for the hypusinated form of eIF5A in higher organisms, it is tempting to speculate that eIF5A and its hypusine modification pathway have evolved along with the increase in polyproline-containing proteins with critical functions in higher eukaryotes. The translation promoting activity of EF-P or eIF5A may not be limited to PPG or PPP motifs. Additional motifs including AAP or YIRYIR were identified as additional motifs whose translations are enhanced by EF-P in bacteria [57]. Future investigations are warranted to establish additional target motifs of eIF5A, the precise mechanism of eIF5A in promoting translation of these motifs and to establish their roles in eukaryotic evolution.

## Supporting Information

Figure S1 **Frequencies of various amino acids in different orthologs pools.**
(TIF)

Table S1 **The total numbers of PPP/PPG motif, ORFs, amino acids and the frequencies of PPP/PPG motifs in the whole proteome of each organism.**
(XLSX)

Table S2 ***M. cuprina*** **proteins with ≥1 PPP units.**
(XLSX)

Table S3 ***M. cuprina*** **proteins with ≥1 PPG units.**
(XLSX)

Table S4 ***E. coli*** **proteins with ≥1 PPP units.**
(XLSX)

Table S5 ***E. coli*** **proteins with ≥1 PPG units.**
(XLSX)

Table S6 ***G. lambia*** **proteins with ≥1 PPP units.**
(XLSX)

Table S7 ***G. lambia*** **proteins with ≥1 PPG units.**
(XLSX)

Table S8 **Yeast proteins with ≥1 PPP units.**
(XLSX)

Table S9 **Yeast proteins with ≥1 PPG units.**
(XLSX)

Table S10 **A.** ***thaliana*** **proteins with ≥1 PPP units.**
(XLSX)

Table S11 **A.** ***thaliana*** **proteins with ≥1 PPP units.**
(XLSX)

Table S12 ***D. melanogaster*** **proteins with ≥1 PPP units.**
(XLSX)

Table S13 ***D. melanogaster*** **proteins with ≥1 PPG units.**
(XLSX)

Table S14 ***M. musculus*** **proteins with ≥1 PPP units.**
(XLSX)

Table S15 ***M. musculus*** **proteins with ≥1 PPG units.**
(XLSX)

Table S16 ***H. sapiens*** **proteins with ≥1 PPP units.**
(XLSX)

Table S17 ***H. sapiens*** **proteins with ≥1 PPG units.**
(XLSX)

Table S18 **List of 76 genes of** ***S. cerevisiae*** **encoding highest proline repeats and their functions.**
(XLSX)

Table S19 **257** ***S. cerevisiae*** **orthologous genes of pool 1.**
(XLSX)

Table S20 **148 human genes encoding PPP at high frequency (>0.01).**
(XLSX)

Table S21 **80 human genes encoding PPG at the frequency (>0.01).**
(XLSX)
