# Supplementary figures and images for: Genome-Wide Analyses and Functional Classification of Proline Repeat-Rich Proteins: Potential Role of eIF5A in Eukaryotic Evolution
Source: PLoS One. 2014 Nov 3;9(11):e111800. doi: 10.1371/journal.pone.0111800 (PMC4218817; doi:10.1371/journal.pone.0111800)

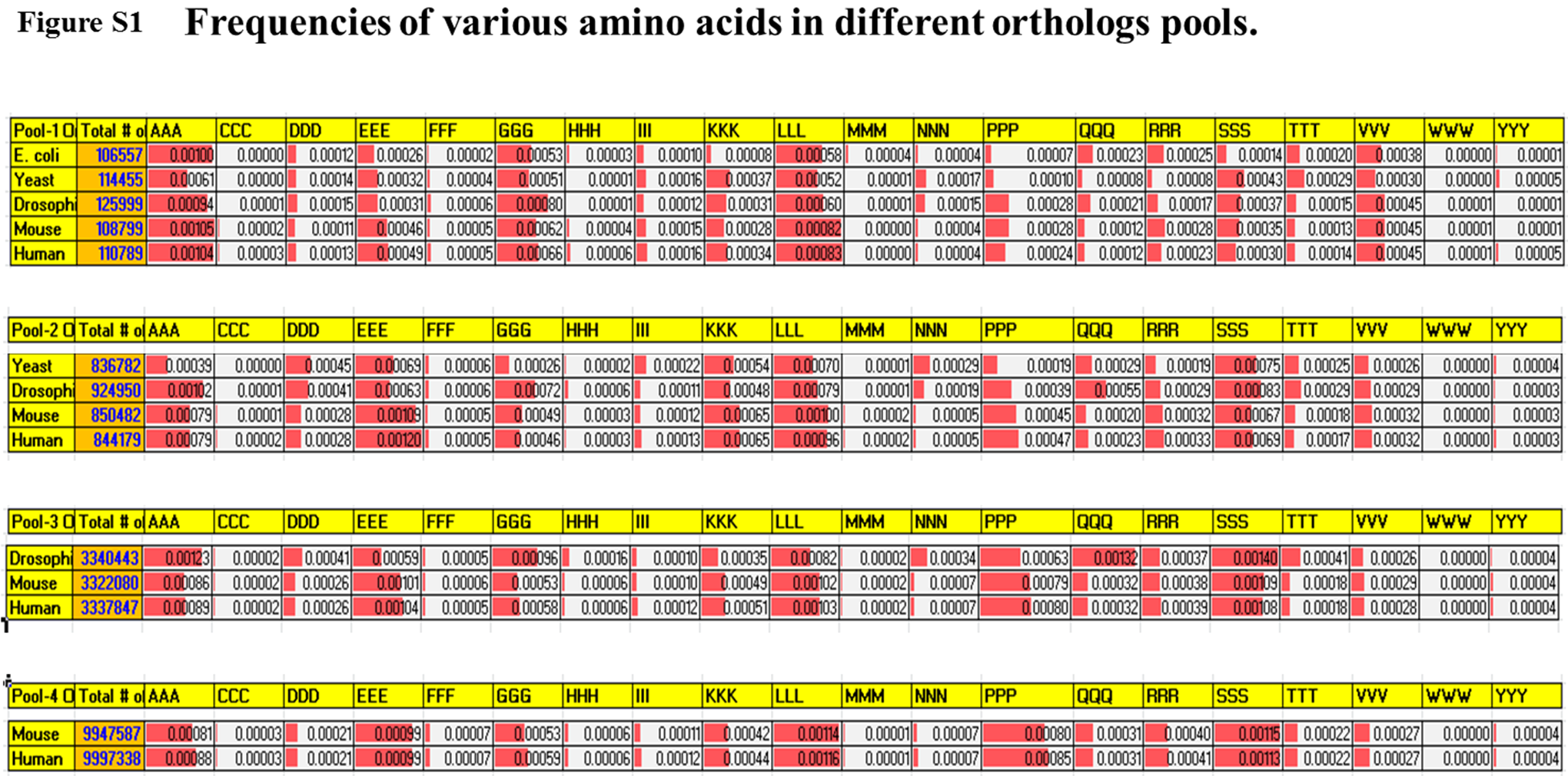

Supplement: Figure S1 — Frequencies of various amino acids in different orthologs pools. (TIF) [file pone.0111800.s001.tif]
